# Iliopsoas Muscle Weakness as a Key Diagnostic Marker in HTLV-1-Associated Myelopathy/Tropical Spastic Paraparesis (HAM/TSP)

**DOI:** 10.3390/pathogens12040592

**Published:** 2023-04-13

**Authors:** Eiji Matsuura, Satoshi Nozuma, Mika Dozono, Daisuke Kodama, Masakazu Tanaka, Ryuji Kubota, Hiroshi Takashima

**Affiliations:** 1 Department of Neurology and Geriatrics, Kagoshima University Graduate School of Medical and Dental Sciences, Kagoshima 890-8520, Japan; 2 Division of Neuroimmunology, Joint Research Center for Human Retrovirus Infection, Kagoshima University, Kagoshima 890-8520, Japan

**Keywords:** HAM/TSP, muscle weakness, muscle involvement, myopathic feature, HTLV-1

## Abstract

Human T-cell leukemia virus-1 (HTLV-1)-associated myelopathy/tropical spastic paraparesis (HAM/TSP) is a slowly progressive neurological disease that arises from HTLV-1 infection. Pathologically, the condition is characterized by diffuse myelitis, which is most evident in the thoracic spinal cord. Clinical manifestations of the infectious disease, HAM/TSP, are empirically known to include weakness of the proximal muscles of the lower extremities and atrophy of the paraspinal muscles, which is characteristic of the distribution of disturbed muscles usually seen in muscular diseases, except that the upper extremities are almost normal. This unique clinical presentation is useful information for physicians and physical therapists involved in diagnosing and rehabilitating patients with HAM/TSP, as well as critical information for understanding the pathogenesis of HAM/TSP. However, the precise pattern of muscle involvement in this condition has yet to be reported. The purpose of this study was to identify the muscles affected by HAM/TSP in order to understand the pathogenesis of HAM/TSP as well as to aid in the diagnosis and rehabilitation of HAM/TSP. A retrospective review of medical records was conducted on 101 consecutively admitted patients with HAM/TSP at Kagoshima University Hospital. Among 101 patients with HAM/TSP, all but three had muscle weakness in the lower extremities. Specifically, the hamstrings and iliopsoas muscle were the most frequently affected in over 90% of the patients. Manual muscle testing (MMT) revealed that the iliopsoas was the weakest of the muscles assessed, a consistent feature from the early to advanced stages of the disease. Our findings demonstrate a unique distribution of muscle weakness in HAM/TSP, with the proximal muscles of the lower extremities, particularly the iliopsoas muscle, being the most frequently and severely affected.

## 1. Introduction

Human T-lymphotropic virus type 1 (HTLV-1)-associated myelopathy/tropical spastic paraparesis (HAM/TSP) is an infectious neurological disease in which HTLV-1 infection causes chronic inflammation of the spinal cord and gradual loss of function in the lower extremities [1,2]. Symptoms of HAM/TSP can be divided into four major categories: motor dysfunction and spastic paraparesis; sensory dysfunction, dysesthesia, and mild hypoesthesia and pain in the lower extremities; autonomic dysfunction, dysuria, and loss of sweating; and complications such as back pain, uveitis, and Sjogren’s syndrome [3]. The symptoms in all four of these categories are often present in combination, but patients do not develop all four categories of symptoms at once; some patients may have only frequent urination or leg cramps for a long period of time, and in rare cases, some may develop gait disturbance after self-catheterization. The major route of HTLV-1 infection is mother-to-child transmission through breast milk, and therefore most infected patients are infected during infancy. However, HTLV-1 infection rates vary by age of birth, gender and country, and have gradually decreased over time. Although women are more likely to be infected than men in older generations of Japanese born before the 1940s [4], on the contrary, men are known to be more infected than women in older Indigenous Australians [5], and the cause is unknown. The cause of the decline in the infection rate with generations long before infection prevention measures began to be taken is not clear, but a decrease in the duration of breastfeeding due to lifestyle changes is thought to be one of the causes. The average age of onset of HAM/TSP is around 50 years old, and the male-to-female ratio is about 3:1, while it is known that HAM/TSP develops relatively early after a blood transfusion or organ transplantation [6,7,8].

The clinical symptoms of HAM/TSP are consistent with the spread of inflammation in the spinal cord. Histopathologically, inflammatory cells diffusely spread longitudinally and transversely throughout the spinal cord, mainly in the middle and lower thoracic spinal cord, with no localized lesions as seen in multiple sclerosis or neuromyelitis optica [9,10]. Degeneration of the spinal cord white matter is symmetrical and diffusely spreading, with more intense degeneration, especially in the anterolateral and medial posterior columns, where long path demyelination is particularly detectable by LFB. Pyramidal signs indicative of a long tract disturbance, such as increased deep tendon reflexes in the extremities, spasticity in the lower extremities, pathological reflexes, and loss of abdominal wall reflexes, are identified in patients with HAM/TSP, even in the early stages of the disease with no subjective symptoms.

On the other hand, muscle strength in the upper limbs remains almost undiminished even when both lower limbs are completely paralyzed, compensating for the loss of function in the lower limbs. The difference in the severity of symptoms between the upper and lower extremities is thought to be because the inflammatory site within the spinal cord is below the thoracic spinal cord. Empirically, HAM/TSP often manifests as proximal muscle weakness in the lower extremities [11,12] with leg spasticity [13]. In addition, paraspinal muscle involvement has been noted early in the disease, and rehabilitation of the trunk and lumbar region is important for patients with HAM/TSP [14]. While muscle weakness of the proximal muscles of the lower extremities and atrophy of the paraspinal muscles are clinical features of HAM/TSP and are useful information for physicians, the cause of this weakness is completely unknown. In addition, which muscles of the body are most frequently affected and which muscles of the body are weakest in HAM/TSP patients is only empirically known and has never been reported in the past. This study was performed to determine the distribution and strength of affected muscles in patients with HAM/TSP in Kagoshima, Japan, an area with particularly high rates of HTLV-1 infection.

## 2. Material and Methods

**Figure 1:**
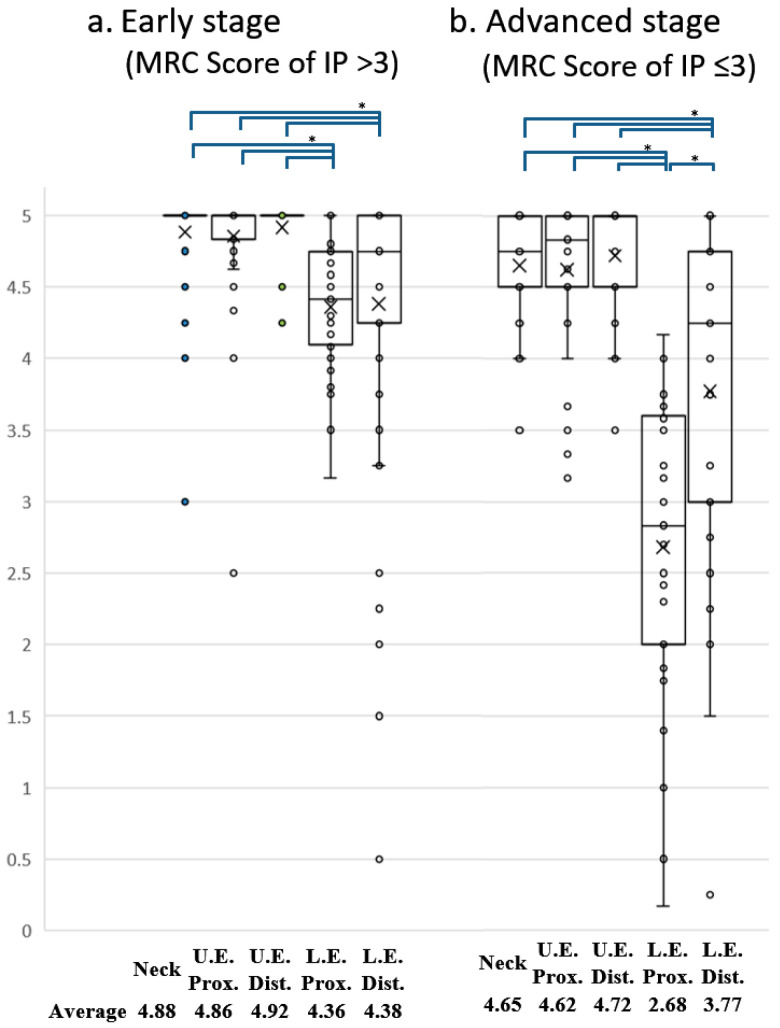
Comparison of MRC score for five body sites in early and advanced stages of HAM/TSP. Box and whisker plots showing the distribution of MRC scores for muscles assessed by the Manual Muscle Test (MMT). The averaged MRC scores for the five body sites were compared between early (**a**) and advanced (**b**) stages of the disease. The × symbol represents the mean value for each group. Muscle strength in the proximal part of the lower extremity was significantly lower than that in any other part of the body in HAM/TSP patients consistently from early (**a**) to advanced (**b**) stages of the disease. A significant difference in strength among the five body parts was detected in both early and advanced stages using the Quade test (*p* < 0.05). Post hoc comparisons using Bonferroni-corrected Wilcoxon signed-rank sum tests showed significant differences ( * ) in all combinations of the two groups indicated at the top of the graph (*p* < 0.005).

**Figure 2:**
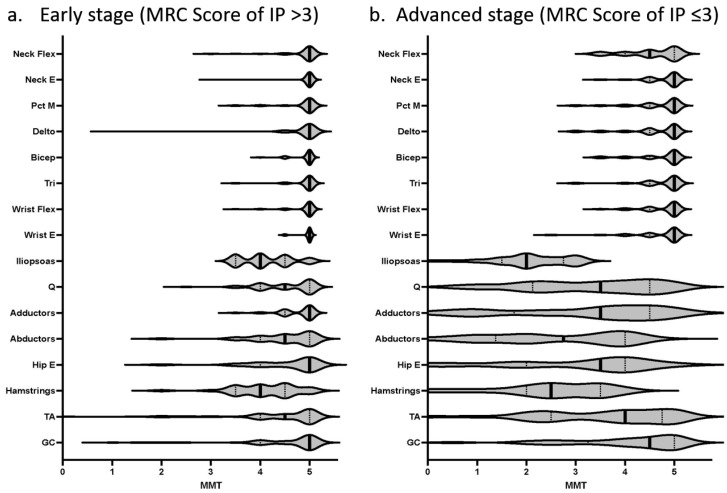
Comparison of MRC scores for all muscles assessed by MMT in HAM/TSP patients. MRC scores for all muscles assessed by the Manual Muscle Test (MMT) are illustrated in a violin plot. The iliopsoas muscle was consistently the weakest muscle in HAM/TSP from the early (**a**) to advanced (**b**) stages of the disease. The thigh abductor muscles were weaker than the thigh adductor muscles from the early (**a**) to advanced (**b**) stages of the disease.

We reviewed the medical records of 184 patients with HAM/TSP consecutively admitted to Kagoshima University Hospital in Kagoshima from January 2002 to December 2012. HAM/TSP was diagnosed according to the World Health Organization diagnostic criteria [15]. The clinical variables measured included sex, age, age at onset, and muscle strength. Muscle strength was evaluated with manual muscle testing and assigned a Medical Research Council (MRC) score ranging from 0 to 5 (0: paralyzed, 1: only traces or flickers of muscle contraction are seen or felt, 2: muscle movement is possible in the absence of gravity, 3: muscle movement is possible against gravity, 4: muscle strength is reduced but the movement against resistance is possible, and 5: normal strength). The MRC scores were collected from the medical records for five muscle regions: neck, proximal upper extremity, distal upper extremity, proximal lower extremity, and distal lower extremity. The muscles evaluated in the five sites were the cervical flexors (neck flexion) and extensors (neck extension) in the neck; pectoralis major (shoulder adduction), deltoids (shoulder abduction), biceps brachii (elbow flexion), and triceps brachii (elbow extension) in the proximal upper extremity; carpal flexors (wrist flexion) and extensors (wrist extension) in the distal upper extremity; iliopsoas (hip flexion), adductor brevis (hip adduction), abductor brevis (hip abduction), quadriceps brachii (knee extension), and hamstrings (knee flexion) in the proximal lower extremity; and tibialis anterior (ankle dorsiflexion) and gastrocnemius (ankle plantar flexion) in the distal lower extremity. The abdominal and back muscles were excluded from evaluation because many patients did not undergo these analyses. To determine which of the evaluated muscles were most frequently impaired, the frequency of patients who were impaired for each of the evaluated muscles was determined, and the average MRC score for those muscles was also calculated (Table 1). To determine which parts of the body were most severely impaired, we calculated the mean MRC score for each body part and compared the mean score values for the five parts (Figure 1). Differences in the MRC scores among the five body sites were tested with the Quade test with an abstention rate of 5%, and differences between the two sites were tested with the Wilcoxon signed rank sum test with Bonferroni correction (*p* < 0.005). Finally, the distribution of MRC scores for all muscles is shown in Figure 2 to facilitate an understanding of the distribution trend of muscle weakness in HAM/TSP patients. (Figure 2).

## 3. Results

In total, 184 patients with HAM/TSP were hospitalized during the study period, some of whom were hospitalized multiple times, including for purposes other than treatment of neurological disease; 1 patient was hospitalized five times, 2 patients were hospitalized four times, 4 patients were hospitalized three times, and 22 patients were hospitalized two times. We collected MRC scores as most recently assessed by a neurologist if the patient had been hospitalized more than once. Finally, the MRC scores were evaluated for a total of 144 patients. Of the 144 patients with HAM/TSP, 43 were hospitalized for complications and excluded because they did not undergo muscle strength evaluation; finally, 101 patients who underwent muscle strength evaluation were analyzed in this study (Table 1a). The 101 patients ranged in age from 14 to 83 years (average, 61.4 years) and comprised 30 male and 71 female patients with a sex ratio of 3:7 (Table 1a). Age at onset ranged from 13 to 76 years (average, 50.4 years). The duration of the disease ranged from a few months to 49 years (average, 10.9 years). When rapid progression was defined by the deterioration of Osame’s motor disability score by 3 points or more than 3 grades within 2 years, 23 (23%) patients presented with rapid disease progression [16]. Of the 101 patients with HAM, 3 (3.0%) had no muscle weakness and showed spastic gait only (Table 1b). Muscle weakness was most frequently observed in knee flexion (52/57, 92.1%) and hip flexion (92/101, 91.1%). All six of the proximal lower extremity muscles evaluated were among the top seven most frequently impaired muscles in the entire body, and the strength of those six muscles was also among the top seven weakest muscles in the entire body (Table 1b). Interestingly, of the 92 patients who showed weakness of hip flexion, 33 (33/92, 35.5%) had normal dorsiflexion and plantar flexion of the ankle joint, meaning that in many cases the distal muscles were not affected at all. These results showed that HAM/TSP most frequently affected the proximal muscles of the lower extremities and most severely weakened them and that only the proximal muscles of the lower extremities were frequently affected; this possibly occurred because of the early stage of the disease, which may have resulted in fewer muscles being affected. To determine whether a similar trend is seen in patients with advanced HAM/TSP, the muscle strength of each muscle was assessed in patients with an iliopsoas (IP) MRC score of 3 or less, corresponding to the level of inability to lift the leg by oneself (Table 2). In the advanced stages of HAM/TSP (MRC score of IP ≤3), three proximal muscle groups of the lower extremities, hip flexors, hip abductors, and knee flexors showed muscle weakness in all cases (100%) and an average MRC score of less than 3, while there were large differences in muscle strength among these three muscle groups, with the weakest being the iliopsoas muscle with an MRC score of 2.08. Similar to the trend seen in early HAM/TSP, the ankle flexors and dorsiflexors, the distal muscles among the lower extremity muscles, were less frequently affected and to a lesser degree in the advanced stage. Even among the 33 patients with an MRC score of ≤3 and an inability to lift their legs, 7 (7/33, 21.2%) had normal dorsiflexion and plantar flexion of the ankle joint.

Our results revealed that 15.8% of the patients had muscle weakness only in the proximal part of the lower extremity. On the other hand, few patients showed muscle weakness in only one other region. These results indicate that the proximal muscles of the lower extremities are frequently and relatively specifically affected in HAM/TSP patients. Next, to determine whether the degree of muscle weakness in the proximal muscles of the lower extremity is also significantly weaker, a statistical analysis was performed to compare muscle strength in five regions: the proximal and distal parts of the upper extremity, the proximal and distal parts of the lower extremity, and the neck. The results revealed that the proximal muscle group of the lower extremity was significantly weaker than any other region (data not shown). However, since the distribution and extent of muscle weakness may not coincide between the early and advanced stages of the disease, we compared the muscle strength of the five regions separately in the early and advanced stages of HAM/TSP. As a result, we found that the proximal muscles of the lower limbs were significantly weaker than those of other regions in HAM/TSP patients not only in the early stage of the disease but also in the advanced stage (Figure 1a,b).

Paraspinal muscle atrophy is not uncommon in patients with HAM/TSP. Although the paraspinal muscles could not be evaluated, 27 (28.4%) of the 95 patients presented with weakness of neck flexion, and 12 (13.2%) of the 91 patients presented with weakness of neck extension (Table 1b). All 12 patients with weak neck extensors had weak neck flexors. This means that the neck flexor muscles were more frequently affected, and patients with weak neck flexor muscles (27/95) included 12 patients with weak neck extensor muscles. Interestingly, the neck flexors were more frequently affected than any other upper extremity muscle (Table 1b), and their strength was also weaker than any other upper extremity muscle. On the other hand, the neck extensor was affected less frequently and none of the patients had dropped-head symptoms. In addition to these features of muscle weakness, an empirically known clinical manifestation of HAM/TSP is that the thigh abductor muscle group is weaker than the thigh adductor muscle group among the proximal muscles of the lower extremity, and this feature was consistently observed from the early to advanced stages of the disease in this study (Table 1b and Table 2, Figure 2).

## 4. Discussion

In this study, we investigated which muscles are affected in patients with HAM/TSP and found that the proximal muscles of the lower extremities, especially the iliopsoas and hamstrings muscles, are consistently the most frequently affected and the iliopsoas muscle was consistently the weakest muscle from the early to advanced stages of the disease. When considering degenerative diseases of the nervous system, according to the established traditional clinical method of weakness distribution, central lesions are thought to affect the extensor muscles more than the flexors in the upper extremity and the flexors more than the extensors in the lower extremity [17]. Hence, weakness of the iliopsoas muscle and hamstrings may be a natural consequence. However, the site of inflammation should vary slightly from patient to patient when organ damage is caused by infection. It is interesting to note that there is a common pattern in the distribution of muscle weakness in HAM patients. Our results also showed a clear difference between the proximal and distal muscles with preserved ankle flexor and extensor muscles. This unique clinical feature resembles the symptoms of myopathy, and physicians who see a patient with HAM/TSP for the first time are expected to be puzzled by these symptoms. HAM/TSP has been suggested to be associated with myositis since its discovery [18,19,20,21,22]. Epidemiological studies have shown that HTLV-1 infection is common in patients with polymyositis, and cases of HAM/TSP complicated by sporadic inclusion body myositis have also been reported [23,24]. Examination of muscle biopsies in patients with HAM/TSP reveals necrotic fibers with cellular infiltration regardless of the creatine kinase concentration in the blood [19]. A possible reason why many complicated cases of myositis have been reported is that patients with HAM/TSP present with weakness of the proximal muscles, causing physicians to suspect muscle disease. Further studies are needed to clarify the association between HTLV-1 and muscle disease. Even without concomitant muscular disease, past studies have shown that patients with HAM/TSP have proximal muscle weakness [11,12,13,25,26], and magnetic resonance imaging studies suggest the same [27]. In patients with hereditary spastic paraparesis unrelated to HTLV-1 infection, proximal muscle weakness is not common [28], whereas, in patients with bulbospinal muscular atrophy and some hereditary peripheral neuropathies, proximal muscle weakness is even a hallmark of the disease [29,30]. Does the presence of certain muscles susceptible to damage in patients with HAM/TSP mean that certain parts of the spinal cord are more prone to inflammation caused by HTLV-1 infection? Previous histological studies have revealed an advanced inflammatory change in the middle to lower portion of the thoracic cord and mild inflammatory change in the cervical and lumbosacral cord. Lesions are symmetrically distributed mainly in the lateral column and to a lesser extent in the inner portion of the posterior columns; they are also present in the central gray matter [9,10]. Takezawa et al. [26] performed a study of rehabilitation for muscle weakness of the trunk and proximal muscles of the lower extremities and reported that the weakness in patients with HAM/TSP can be explained at the level of the spinal cord segments, given that the innervation of the erector spinae muscles is distributed in the lower thoracic segments with trunk extensors from Th6 to Th11 and trunk flexors from Th7 to Th11. However, patients with HAM/TSP usually have stronger hip adductor muscles (L2-4) and weaker hip abductor muscles (L5S1), and the present study also confirms that the abductor muscle group is weaker than the adductor muscle group, suggesting that there are factors other than spinal segmental level. This feature may be the result of spasticity due to a disturbance in the long pathway of the spinal cord. A recent study revealed the most frequently spastic muscles in patients with HAM/TSP [13]. There is no apparent correlation between the degree of spasticity found in that study and the muscle weakness shown in our study, and it does not appear that strength is necessarily preserved in the more spastic muscles.

Paraspinal muscle atrophy is frequently noted in patients with HAM/TSP, and electromyography has confirmed paraspinal muscle abnormalities [14]. Atrophy of the paraspinal muscles may be the reason for the high prevalence of low back pain in patients with HAM/TSP [31], as well as in patients with severe thoracic kyphosis and lumbar lordosis [32]. In the present study, we evaluated neck muscle strength as one of the trunk muscles and found that 28.4% of patients had neck flexion muscle weakness. The frequency of cervical muscle involvement was low, but it may indicate atrophy of the paraspinal muscles, and further study (including imaging) is needed to determine the relationship between the two. Finally, physicians who treat HAM may feel uncomfortable with the high frequency of upper extremity muscle weakness (20%) shown in this study. These data were based on the information obtained from inpatient neurologic examination forms; the attending physician may have assessed upper extremity muscle strength with the patient sitting on the bed despite the fact that HAM/TSP is a disease that weakens the trunk muscles. The upper extremity weakness may include examiner error.

## 5. Conclusions

Patients with HAM/TSP showed a unique distribution of muscle weakness in the proximal muscles, particularly in the iliopsoas muscle.

## Figures and Tables

**Table 1:** Clinical characteristics and muscle involvement in HAM/TSP patients.

a. Patient Characteristics				
Age, Years				61.4 (14–83)
Sex, male/female				30/71
Age at disease onset, years				50.4 (13–76)
Duration of disease, years				10.9 (0.3–49)
Rapid progression				23 (23%)
Data are presented as average (range), *n*, or *n* (%).				
**b. Muscles susceptible to injury from HAM/TSP (%)**				
Patients presenting with no muscle weakness but with spastic gait: 3/101 patients (3.0%)				
**Manual muscle testing**	**Name of muscles**	**Segment level** **in spinal cord**	**Average MRC score (0–5)**	**Percentage of impairment (%)**
Knee flexion	Hamstrings	L5S1	3.50	91.2
Hip flexion	Iliopsoas	L3L4	3.46	91.1
Hip abduction	Gluteus medius +	L5S1	3.74	73.2
Knee extension	Quadriceps	L3L4	4.11	63.3
Hip extension	Gluteus maximus	L5S1	3.92	63.2
Ankle dorsiflexion	Tibialis anterior	L5	4.06	59.0
Hip adduction	Leg adductors	L234	4.13	57.1
Ankle plantar flexion	Gastrocnemius +	S1	4.32	47.4
Neck flexion	Neck flexors	C1-5	4.74	28.4
Shoulder abduction	Deltoid	C5	4.76	26.0
Shoulder adduction	Pectoralis major	C5-Th1	4.78	23.5
Elbow flexion	Biceps brachii	C5	4.83	22.8
Elbow extension	Triceps brachii	C7	4.83	21.6
Wrist flexion	Wrist flexors	C7-8	4.84	21.4
Wrist extension	Wrist extensors	C678	4.86	18.6
Neck extension	Neck extensors	C1-8	4.90	13.2
**c. Affected part of the body (%)**				
Neck	Upper extremities		Lower extremities	
	U.E. Prox.	U.E. Dist.	L.E. Prox.	L.E. Dist.
28.4% (27/95)	43.6% (44/101)		96.0% (97/101)	
	40.6% (41/101)	22.4% (22/98)	95.0% (96/101)	62.0% (62/100)
Neck only	U.E.Prox. only	U.E.Dist. Only	L.E.Prox. Only	L.E.Dost. Only
0	1.0% (1/101)	0	15.8% (16/101)	1% (1/100)

+: Other muscles are evaluated together by manual muscle testing (MMT).

**Table 2:** Muscle strength (MRC score) in 33 patients with advanced HAM/TSP *.

Manual Muscle Testing	Name of Muscles	Segment Level in Spinal Cord	Average of MRC Score (0–5)	Percentage of Impairment (%)
Hip flexion	Iliopsoas	L3L4	2.08	100.0
Knee flexion	Hamstrings	L5S1	2.55	100.0
Hip abduction	Gluteus medius +	L5S1	2.61	100.0
Hip extension	Gluteus maximus	L5S1	3.03	93.3
Hip adduction	Leg adductors	L234	3.16	86.4
Knee extension	Quadriceps	L34	3.28	87.5
Ankle dorsiflexion	Tibialis anterior	L5	3.55	75.8
Ankle plantar flexion	Gastrocnemius +	S1	4.00	63.6
Neck flexion	Neck flexors	C1-5	4.55	51.6
Shoulder abduction	Deltoid	C5	4.59	43.8
Shoulder adduction	Pectoralis major	C5-Th1	4.65	39.1
Wrist extension	Wrist extensors	C678	4.69	34.4
Elbow flexion	Biceps brachii	C5	4.70	33.3
Elbow extension	Triceps brachii	C7	4.74	33.3
Wrist flexion	Wrist flexors	C78	4.76	33.3
Neck extension	Neck extensors	C1-8	4.79	32.1

* Advanced HAM/TSP is defined as a condition in which the MRC score of iliopsoas muscle strength is 3 or less. +: Other muscles are evaluated together by manual muscle testing (MMT).

## Data Availability

Not applicable.
